# The Impact of Urine-Sample HPV Testing on the Effectiveness of Screening for Cervical Cancer: An Umbrella Review

**DOI:** 10.3390/cancers16122244

**Published:** 2024-06-17

**Authors:** Wojciech Miazga, Tomasz Tatara, Katarzyna Wnuk, Mariusz Gujski, Jarosław Pinkas, Urszula Religioni

**Affiliations:** 1 School of Public Health, Centre of Postgraduate Medical Education of Warsaw, Kleczewska 61/63, 01-826 Warsaw, Poland; w.miazga@aotm.gov.pl (W.M.);; 2 Department of Public Health, Faculty of Health Sciences, Medical University of Warsaw, Żwirki i Wigury 61, 02-091 Warsaw, Poland

**Keywords:** cervical cancer, prevention, urine sample, human papillomavirus, HPV, HPV testing

## Abstract

**Simple Summary:**

Our purpose was to assess the impact of urine-sample HPV (human papillomavirus) testing on the effectiveness of screening for cervical cancer. We also assessed the acceptability of the self-collection of urine samples for HPV screening tests. Our analysis took into account research results published in systematic reviews of the available literature on the topic under study. Of the 1869 articles found in this area, we included 5 studies that met the criteria we assessed. We discovered that the sensitivity and specificity for the detection of any HPV in first-void urine samples were 87% [95% CI: (0.74; 0.94)] and 89% [95% CI: (0.81; 0.93)]. Moreover, participants in the analyzed studies had indicated that they felt comfortable with urine testing. We therefore conclude that the detection of HPV infection in first-void urine samples may have great diagnostic significance. The use of this method in commonly available screening tests could significantly increase patients’ willingness to participate in research.

**Abstract:**

Background: The aim of the study was to evaluate the impact of urine-sample HPV (human papillomavirus) testing on the effectiveness of screening for cervical cancer. Methods: The analysis was based on the results of a systematic review. Secondary studies were searched in the following medical databases: Medline, Embase, and the Cochrane Library. The results of the statistical tests presented in the article originate from research conducted by the authors of the included articles. Results: From a total of 1869 citations, 5 studies were included in this review. Sensitivity and specificity for the detection of any HPV from first-void urine samples were 87% [95% CI: (0.74; 0.94)] and 89% [95% CI: (0.81; 0.93)], respectively. Moreover, participants in the analyzed studies had indicated that they felt comfortable with urine testing. Conclusions: The development of methods to detect HPV infection in first-void urine samples and the application of this sampling method in widely available screening tests could significantly increase patients’ willingness to participate in testing.

## 1. Introduction

According to data from the World Health Organization (WHO) and the International Agency for Research on Cancer (IARC) as presented for the year 2022 on the Global Cancer Observatory (GCO) interactive web-based platform presenting global cancer statistics, cervical cancer was the fourth most common cancer in women (662,301 cases worldwide) while ranking eighth among the most prevalent cancers regardless of patient gender. Cervical cancer was also the fourth most common cause of cancer deaths in women (348,874 deaths) and the ninth most common cause of death from all cancers regardless of patient gender [1].

Human papilloma virus (HPV) infection is considered to be a prerequisite for the development of cervical cancer [2]. 

Accordingly, in May 2020, the WHO published the *Global strategy to accelerate the elimination of cervical cancer as a public health problem*, calling on all institutions involved in cervical-cancer prevention worldwide to take steps to eliminate cervical cancer as a population problem, i.e., to reduce the incidence to ≤4 cases/100,000/year by the end of the current century. The first step of the strategy involves the achievement of the minimum goals, referred to by the acronym “90-70-90”, by 2030 [3].

Despite the development of effective methods for the early detection of cervical cancer (such as cytology, testing for oncogenic HPV subtypes, or a combination of the above) [4], one of the problems associated with screening is the low turnout of female patients, which translates into a low effectiveness of screening programs. In 2022, our team carried out an umbrella review to assess the impact of allowing women to self-collect vaginal samples for HPV testing on the effectiveness of early-detection screening for cervical cancer [5]. In this article, we present the results of a study analyzing the impact of using the method for the detection of HPV infection in urine samples, which has the potential to increase the CC screening reporting rates by virtue of the ease of sample collection. Therefore, the aim of the review was to find an answer to the question of whether HPV testing using urine samples would affect the effectiveness of cervical-cancer screening.

## 2. Materials and Methods

The clinical analysis was based on the results of studies identified from a systematic review performed according to the following scheme:

- determination of the criteria for the inclusion of studies in the review;
- development/verification of search strategies;
- the query/re-query of medical information sources;
- identification of full-text reports potentially useful in the clinical analysis;
- selection of studies based on the inclusion criteria;
- result processing;
- qualitative synthesis involving the analysis of the statistical and clinical significance of the results of the studies included in the review.

The search for clinical trials was based on a detailed pre-developed protocol. The systematic review was conducted in accordance with the Cochrane Collaboration guidelines [6], taking into the account the criteria for the inclusion of studies in the review, the search strategy, the selection process, and the planned methodology of analysis.

Clinical trials included in the analysis met the following criteria: • population: adult female population; • intervention: HPV testing performed using a urine sample; • comparators: unlimited (all possible); • methodology: meta-analyses of randomized and/or observational studies and systematic reviews of randomized and/or observational studies; and • endpoints: effectiveness, including sensitivity and specificity of CC screening tests, CC screening reporting rates, and acceptance of CC screening sampling by the study population.

The following medical information sources were queried for secondary studies: Medline (via PubMed), Embase (via Ovid), and the Cochrane Library. The last query was performed on 6 February 2024 as per the search strategy summary provided in the Supplementary Materials. 

**Figure 1:**
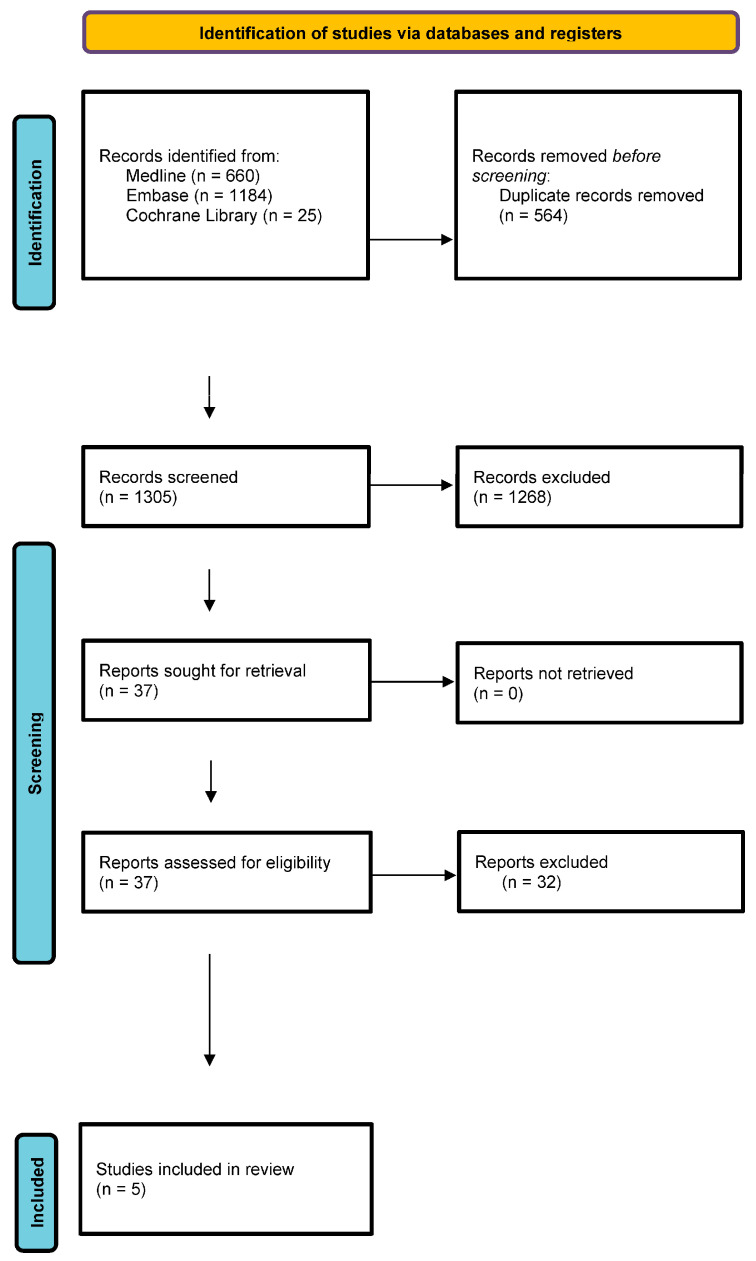
The PRISMA flow diagram.

At all stages of the systematic review, the selection of studies was independently performed by two analysts (W.M. and K.W.). Discrepancies were resolved by means of a consensus with a third independent analyst (T.T.). The most common reasons for the exclusion of studies from the analysis were issues related to the intervention (failure to include a urine-sample-based method for HPV detection) and methodology (failure to properly describe the materials and methods or an improper synthesis of review results). The stages of the study selection are shown in Figure 1. For the list of publications included and excluded from the review, see the Supplementary Materials.

The assessment of the quality and the risk of error for the secondary studies included in the analysis were evaluated by reviewing the key domains of the AMSTAR2 systematic review assessment tool [7]. This tool is capable of identifying publications with the highest quality. According to the rules, a publication receives the highest rating when we receive positive answers to all questions. A noticeable single deficiency in a domain causes a systematic review to be downgraded. One deficiency results in the rating being downgraded to the “low”-quality category, while two or more deficiencies result in a “critically low” rating. This type of quality assessment was carried out by two analysts (K.W. and W.M.). Any discrepancies were resolved by a third independent analyst (T.T.) by a consensus. The protocol for the systematic review of our study was not registered with PROSPERO. Detailed information is provided in the Supplementary Materials. The results of the statistical analyses conducted by the authors of the primary studies were used in the secondary research. The results of each publication were presented separately.

### Limitations of the Review

Only English-language publications were included in the review. The search was narrowed to publications from the last 10 years (1 January 2014–6 February 2024). Studies included in the secondary scientific evidence encompassed an ethnically and geographically diverse population. Significant limitations of this review were the lack of a well-defined uniform method to detect HPV in urine and the heterogeneity between studies.

## 3. Results

The following five studies met the criteria for inclusion in the systematic review (n = 5: Jordaens, et al. 2023, Cho, et al. 2022, Bober, et al. 2021, Nishimura, et al. 2021, and Pathak, et al. 2014 [8,9,10,11,12]):

- Jordaens, et al. 2023: a systematic review of 924 publications describing the detection of biomarkers in urine for oncological purposes [8];
- Cho, et al. 2022: a meta-analysis of 21 observational studies that evaluated the diagnostic accuracy of the urinary HPV screening test for cervical intraepithelial neoplasia (CIN2) compared with the cervical HPV screening test [9];
- Bober, et al. 2021: a meta-analysis of 15 observational studies assessing the diagnostic accuracy of HPV screening in urine compared with cervical screening for HPV [10];
- Nishimura, et al. 2021: a systematic review of 72 cross-sectional studies that examined the values and preferences for the self-collection of HPV samples [11];
- Pathak, et al. 2014: a meta-analysis of 14 observational studies that assessed the diagnostic accuracy of the urinary HPV screening test compared with the cervical HPV screening test [12].

The results of the secondary studies included in the review are listed below. 

### 3.1. The Use of Urine Samples as Liquid Biopsies in Non-Invasive HPV Testing

The database query returned the Jordaens 2023 paper, consisting of a systematic review of the literature and the identification of 924 studies reporting on the detection of biomarkers in urine for oncological purposes, including 62 studies related to cervical cancer. As shown by the results of the review, four considerations should be taken into account when attempting to make clinical use of HPV detection using urine samples:

- the use of first-void urine collected with appropriately designed devices;
- the use of a preservative to prevent degradation during extraction and storage;
- the use of polymerase chain reaction (PCR)-based assays;
- the collection of a sufficient volume of urine.

The authors of Jordaens, et al. 2023 [8] supported the validity of first-void urine sampling as identified by the results of the Pathak, et al. 2014 [12] study, which was also included in our review, while also pointing to other publications suggesting higher concentrations of hrHPV and human DNA being found in first-void urine. In addition to the collection of first-void urine samples, the authors of the Jordaens 2023 review pointed to the need to refrain from intensive genital washing before collecting the sample from women. Another highlight of the systematic review in question was the fact that, at the time of writing, none of the high-throughput HPV tests identified in the publication had FDA approval or CE marking for use in CC screening from urine samples [8].

### 3.2. Diagnostic Precision of the Detection of HPV Infection from Urine Samples

In the Bober 2021 [10] meta-analysis, which was based on 15 test-of-accuracy studies comparing HPV DNA detection in urine and cervical swab samples, the authors estimated the diagnostic precision of HPV tests performed using first-void urine samples in the identification of three groups of HPVs, i.e., any HPV, high-risk HPV, and HPVs 16 and 18. According to the results of the meta-analysis, the combined sensitivity and specificity for the detection of any HPV from first-void urine samples were 87% [95% CI: (0.74; 0.94)] and 89% [95% CI: (0.81; 0.93)], respectively, while the precision for the detection of high-risk HPV had a sensitivity of 78% [95% CI: (0.70; 0.84)] and a specificity of 89% [95% CI: (0.81; 0.94)]. With regard to the detection of HPVs 16 and 18, tests from first-void urine samples had a combined sensitivity of 77% [95% CI: (0.76; 0.77)] and specificity of 98% [95% CI: (0.98; 0.98)] [10].

The diagnostic precision of HPV tests performed using urine samples in the identification of any HPV, high-risk HPV, and HPVs 16 and 18 was also assessed in the Pathak 2014 [12] meta-analysis. The authors of this publication calculated their results on the basis of 14 test-of-accuracy studies comparing HPV DNA detection in urine and cervical swab samples. According to the results of the meta-analysis, the overall sensitivity and specificity of HPV detection in urine samples were 87% [95% CI: (0.78; 0.92)] and 94% [95% CI: (0.82; 0.98)]. Precision for the detection of high-risk HPV recorded a sensitivity of 77% [95% CI: (0.68; 0.84)] and a specificity of 88% [95% CI: (0.58; 0.97)]. With regard to the detection of HPVs 16 and 18, the urine-sample tests had a combined sensitivity of 73% [95% CI: (0.56; 0.86)] and specificity of 98% [95% CI: (0.91; 1.00)]. The authors of the meta-analysis pointed out that not all of the tests included in the meta-analysis had been performed using first-void urine samples; however, an increase in sensitivity was shown in a meta-regression assessment when first-void urine samples were collected and compared with random or midstream samples (*p* = 0.004) [12].

### 3.3. Diagnostic Precision of the Detection of CIN2+ Using Urine-Sample HPV Tests

The authors of the Cho 2022 meta-analysis [9] compared the diagnostic precision of the detection of cervical intraepithelial neoplasia grade two or worse (CIN2+) from HPV tests using urine vs. vaginal samples collected by clinicians. Based on the 21 observational studies included in the review, the authors’ overall sensitivity of the urine HPV test relative to the clinician-sampled test was calculated to amount to 0.84 [95% CI: (0.78; 0.91)], while the specificity was calculated to amount to 1.06 [95% CI: (1.03; 1.10)]. The results showed a statistically significant 16% reduction in the relative sensitivity of HPV tests performed using urine samples compared with those performed using physician-collected samples, with a slight increase of 6% in relative specificity. 

The combined absolute sensitivity and specificity of detecting CIN2+ using HPV tests performed with urine samples were also calculated by the authors of the Cho 2022 meta-analysis to amount to 79% [95% CI: (0.72; 0.85)] and 55% [95% CI: (0.46; 0.63)], respectively. In comparison, the absolute sensitivity and specificity of detecting CIN2+ using HPV tests from vaginal samples collected by a clinician were 94% [95% CI: (0.90; 0.96)] and 50% [95% CI: (0.40; 0.60)], respectively [9].

The following is a summary of the results of the meta-analyses on the diagnostic precision of HPV tests using urine samples (Table 1).

### 3.4. The Acceptability of Self-Collection of Urine Samples for HPV Screening Tests

As part of the Nishimura 2021 systematic review [11], the authors identified a total of 72 population-based cross-sectional studies on values and preferences in relation to self-sampling for HPV screening tests. Two studies found by the authors addressed the acceptability of vaginal swabs and urine self-sampling for HPV screening tests. As reported by the review authors, 95% of the participants in the first study indicated that they felt comfortable with urine testing, while 82% of the participants declared feeling comfortable with the self-sampling of vaginal swabs. In the second study, 85% of participants with CIN rated the self-sampling procedures (urine and cervical swabs) as easy, with 74% declaring they could imagine taking samples at home on their own. However, the second study did not directly compare the preference regarding the collection of urine samples with the preference regarding the self-sampling of vaginal swabs [11].

## 4. Discussion

Based on the results of the studies included in this systematic review, the sensitivity and specificity of HPV screening tests performed using urine samples were assessed. Publications on the acceptability of urine self-sampling were also analyzed as a factor potentially affecting the future reportability of CC screening.

An umbrella review was conducted by our study team in 2022 to assess the impact of allowing women to self-sample vaginal swabs for HPV tests on the increased effectiveness of screening for the early detection of cervical cancer [5]. The studies identified in 2022 indicated that HPV testing using self-sampled vaginal swabs had acceptable efficiency, with screening enrollment reaching the highest rates when the self-sampling kits were mailed to patients. The inclusion of urine HPV tests in screening programs would further facilitate the method of sample collection to further increase reporting for cervical-cancer screening programs. 

For the purpose of the discussion, an attempt was made to search for current clinical practice guidelines regarding cervical-cancer screening, with a particular focus on HPV screening tests performed using urine samples; however, the sampling method had not been recommended by scientific societies at that point. The self-sampled vaginal swab-based HPV test is a cervical-cancer screening method recognized by organizations such as the American Cancer Society (ACS) [13], the WHO [14], and the United States Preventive Services Task Force (USPSTF) [15].

A recent paper, Zigras 2023 [16], was developed by a working group in collaboration with the Society of Gynecologic Oncology of Canada (GOC), the Society of Canadian Colposcopists (SCC), and the Canadian Partnership Against Cancer, and provides guidance on HPV screening and testing in specific patient populations. With regard to self-sampling, the authors pointed out that a number of studies have shown that a mail distribution of self-sampling kits increased screening rates, while self-sampling combined with face-to-face interactions were more effective for healthcare professionals, nurses, or healthcare workers making home visits [16].

The current status of the worldwide use of HPV self-sampling was described in Serrano 2022 [17]. According to the results of a systematic review of the literature and official documents, supplemented by formal national consultations of the World Health Organization, of the total of 48 countries with HPV-test-based screening programs for cervical cancer, the introduction of self-sampling into these programs was reported by 17 (35%). In nine countries (Albania, Kenya, Guatemala, Honduras, Malaysia, the Netherlands, Peru, Rwanda, and Uganda), vaginal self-sampling for HPV tests was considered to be the main screening option for all women, while in the remaining eight countries (Argentina, Australia, Denmark, Ecuador, Finland, France, Myanmar, and Sweden), self-sampling was recommended for underscreened populations [17]. 

Conclusions from Daponte 2021 [18], an update of a literature review on the feasibility of urine-sample-based HPV tests as part of cervical-cancer screening, indicate that the detection of HPV in urine—due to its low cost, resulting from the bypassing of the need to visit a physician, as well as the non-invasive nature and favorable acceptability profile of the procedure—may become the most promising tool to expand the available options for the further evolution of cervical-cancer-prevention screening programs [18].

Taking all of the above conclusions into account, it seems that a conclusive approach to conduct population-based screening for cervical cancer has not yet been definitively determined. Further research aimed at refining the method of HPV infection detected using urine samples is likely to lead to this method of sample collection being established as a target-population screening strategy. The ease of collecting urine samples combined with minimal pain or discomfort, and thus the high acceptability of this collection method, have the potential to increase reporting for cervical-cancer screening programs in the future. 

## 5. Conclusions

The objective of providing highly effective CC screening tests at a minimum of twice in a lifetime, i.e., at the age of 35 and again at 45, seems only achievable if screening tests performed using samples are collected in a way that is easy and acceptable to women and are widely available. The development of methods to detect HPV infection in first-void urine samples and the application of this sampling method in widely available screening tests could significantly increase patients’ willingness to participate in testing and thus increase the chances of eliminating cervical cancer as a public health problem in the future. The self-collection of urine samples to detect HPV infections promises to be an effective method of screening for the early detection of CC, but more research is needed to develop and officially market validated tests to detect HPV in urine.

## Figures and Tables

**Table 1:** Characteristics and results of the studies on the diagnostic accuracy of HPV screening tests using urine samples.

Author/Year	Sample Collection Method for HPV Test	Endpoint	Endpoint Detection Precision	
			Sensitivity (95% CI) [No. of Studies]	Specificity (95% CI) [No. of Studies]
Cho 2022 (MA) [9]	Total urine sample	Cervical intraepithelial neoplasia grade two or worse (CIN) (2+)	79% (0.72; 0.85) [19 OS].	55% (0.46; 0.63) [19 OS].
Bober 2021 (MA) [10]	First-void urine	Infection with any HPV genotype	87% (0.74; 0.94)] [10 OS]	89% (0.81; 0.93) [10 OS].
		Infection with a high-risk HPV genotype	78% (0.70; 0.84) [12 OS]	89% (0.81; 0.94) [12 OS]
		Infection with HPV genotype 16 or 18	77% (0.76; 0.77) [7 OS]	98% (0.98; 0.98) [7 OS]
Pathak 2014 (MA) [12]	Total urine sample	Infection with any HPV genotype	87% (0.78; 0.92) [14 OS].	94% (0.82; 0.98) [14 OS].
		Infection with a high-risk HPV genotype (16, 18, 31, 33, 35, 39, 45, 51, 52, 56, 58, 59, 68, 73, or 82)	77% (0.68; 0.84) [11 OS].	88% (0.58; 0.97) [11 OS].
		Infection with HPV genotype 16 or 18	73% (0.56; 0.86) [11 OS]	98% (0.91; 1.00) [11 OS]

MA: meta-analysis; CIN: cervical intraepithelial neoplasia; HPV: human papilloma virus; OS: observational study.

## Data Availability

Not applicable.

## Supplementary Materials

The following supporting information can be downloaded at: https://www.mdpi.com/article/10.3390/cancers16122244/s1, File S1: Search strategy: list of studies included and excluded after full-text analysis; AMSTAR2 rate.
