# Supplementary material for: The Impact of Urine-Sample HPV Testing on the Effectiveness of Screening for Cervical Cancer: An Umbrella Review
Source: Cancers (Basel). 2024 Jun 17;16(12):2244. doi: 10.3390/cancers16122244 (PMC11201501; doi:10.3390/cancers16122244)
Supplement: Supplementary file 1 [file cancers-16-02244-s001.zip › cancers-3027760-supplementary.pdf]

**Search strategy Cochrane**

| ID | Keyword | Result |
|---|---|---|
| #1 | (Cervical Cancer):ti,ab,kw | 6313 |
| #2 | (cervical neoplasm*):ti,ab,kw | 4456 |
| #3 | (cervix cancer):ti,ab,kw | 3413 |
| #4 | (cervix neoplasm*):ti,ab,kw | 2251 |
| #5 | (Human Papillomavirus):ti,ab,kw | 2871 |
| #6 | MeSH descriptor: [Human Papillomavirus Viruses] explode all trees | 378 |
| #7 | (Human Papillomavirus):ti,ab,kw | 2871 |
| #8 | (HPV):ti,ab,kw | 3825 |
| #9 | MeSH descriptor: [Papillomavirus Infections] explode all trees | 2208 |
| #10 | (papillomavirus infection*):ti,ab,kw | 2153 |
| #11 | MeSH descriptor: [Papillomaviridae] explode all trees | 1162 |
| #12 | (Papillomaviridae):ti,ab,kw | 736 |
| #13 | #1 OR #2 OR #3 OR #4 OR #5 OR #6 OR #7 OR #8 OR #9 OR #10 OR #11 OR #12 | 11031 |
| #14 | MeSH descriptor: [Human Papillomavirus DNA Tests] explode all trees | 14 |
| #15 | (Human Papillomavirus DNA Test*):ti,ab,kw | 378 |
| #16 | MeSH descriptor: [Urine Specimen Collection] explode all trees | 50 |
| #17 | MeSH descriptor: [Urine] explode all trees | 805 |
| #18 | (Urine):ti,ab,kw | 51163 |
| #19 | #14 OR #15 OR #16 OR #17 OR #18 | 51534 |
| #20 | (selfcollect*):ti,ab,kw | 382 |
| #21 | (self-collect*):ti,ab,kw | 399 |
| #22 | (selfsampl*):ti,ab,kw | 317 |
| #23 | (self-sampl*):ti,ab,kw | 327 |
| #24 | MeSH descriptor: [Mass Screening] explode all trees | 5964 |
| #25 | (Screen*):ti,ab,kw | 100420 |
| #26 | (early diagno*):ti,ab,kw | 36232 |
| #27 | (early test*):ti,ab,kw | 40262 |
| #28 | (early detect*):ti,ab,kw | 16913 |
| #29 | #20 OR #21 OR #22 OR #23 OR #24 OR #25 OR #26 OR #27 OR #28 | 160232 |
| #30 | #19 OR #29 | 203628 |
| #31 | #13 AND #30 with Cochrane Library publication date Between Jan 2014 and Feb 2024, in Cochrane Reviews | 25 |

**Search strategy Medline (via PubMed)**

| ID | Keyword | Result |
|---|---|---|
| #1 | Search: Cervical Cancer[MeSH Terms] | 86,771 |

| | | |
|---|---|---|
| #2 | Search: Cervical Cancer[Title/Abstract] | 61,667 |
| #3 | Search: cervical neoplasm*[Title/Abstract] | 1,435 |
| #4 | Search: cervix cancer[Title/Abstract] | 2,360 |
| #5 | Search: cervix neoplasm*[Title/Abstract] | 4,291 |
| #6 | Search: Human Papillomavirus[MeSH Terms] | 7,730 |
| #7 | Search: Human Papillomavirus[Title/Abstract] | 44,320 |
| #8 | Search: HPV[Title/Abstract] | 53,570 |
| #9 | Search: papillomavirus infections[MeSH Terms] | 43,741 |
| #10 | Search: papillomavirus infection*[Title/Abstract] | 5,137 |
| #11 | Search: Papillomaviridae[MeSH Terms] | 39,006 |
| #12 | Search: Papillomaviridae[Title/Abstract] | 293 |
| #13 | Search: #1 OR #2 OR #3 OR #4 OR #5 OR #6 OR #7 OR #8 OR #9 OR #10 OR #11 OR #12 | 156,358 |
| #14 | Search: Human Papillomavirus DNA Tests[MeSH Terms] | 573 |
| #15 | Search: Human Papillomavirus DNA Test*[Title/Abstract] | 260 |
| #16 | Search: Urine Specimen Collection[MeSH Terms] | 588 |
| #17 | Search: Urine[MeSH Terms] | 38,094 |
| #18 | Search: Urine[Title/Abstract] | 275,021 |
| #19 | Search: #14 OR #15 OR #16 OR #17 OR #18 | 287,459 |
| #20 | Search: selfcollect*[Title/Abstract] | 2,057 |
| #21 | Search: self-collect*[Title/Abstract] | 2,103 |
| #22 | Search: selfsampl*[Title/Abstract] | 1,257 |
| #23 | Search: self-sampl*[Title/Abstract] | 1,326 |
| #24 | Search: Screening[MeSH Terms] | 174,453 |
| #25 | Search: screen*[Title/Abstract] | 1,016,530 |
| #26 | Search: early diagno*[Title/Abstract] | 115,297 |
| #27 | Search: early test*[Title/Abstract] | 826 |
| #28 | Search: early detect*[Title/Abstract] | 88,664 |
| #29 | Search: #20 OR #21 OR #22 OR #23 OR #24 OR #25 OR #26 OR #27 OR #28 | 1,231,001 |
| #30 | Search: #19 OR #29 | 1,492,374 |
| #31 | Search: #13 AND #30 | 29,866 |
| #32 | Search: (((((((systematic[Title]) AND ((Review[Title/Abstract]) OR "Review" [Publication Type]))))))) | 255,620 |
| #33 | Search: ((((((((metaanalysis[Title/Abstract]) OR Meta-Analysis[Title/Abstract]) OR "Meta-Analysis" [Publication Type])))))) | 285,878 |
| #34 | Search: #32 OR #33 | 415,349 |
| #35 | Search: #31 AND #34 | 828 |

| | | |
|---|---|---|
| #36 | Search: #31 AND #34 Filters: from 2014 - 2024 | 660 |

**Search strategy Embase (via Ovid)**

| ID | Keyword | Result |
|---|---|---|
| 1 | exp uterine cervix cancer/ | 119738 |
| 2 | Cervical Cancer.ab,kw,ti. | 85053 |
| 3 | "cervical neoplasm*".ab,kw,ti. | 935 |
| 4 | cervix cancer.ab,kw,ti. | 3432 |
| 5 | "cervix neoplasm*".ab,kw,ti. | 783 |
| 6 | exp Wart virus/ | 45512 |
| 7 | Human Papillomavirus.ab,kw,ti. | 52834 |
| 8 | HPV.ab,kw,ti. | 73264 |
| 9 | exp papillomavirus infection/ | 38435 |
| 10 | "papillomavirus infection*".ab,kw,ti. | 5933 |
| 11 | exp papillomaviridae/ | 61962 |
| 12 | Papillomaviridae.ab,kw,ti. | 377 |
| 13 | 1 or 2 or 3 or 4 or 5 or 6 or 7 or 8 or 9 or 10 or 11 or 12 | 209205 |
| 14 | exp Human papillomavirus DNA test/ | 2835 |
| 15 | "Human Papillomavirus DNA Test*".ab,kw,ti. | 319 |
| 16 | exp urine sampling/ | 25732 |
| 17 | exp urine/ | 94809 |
| 18 | Urine.ab,kw,ti. | 343339 |
| 19 | 14 or 15 or 16 or 17 or 18 | 373387 |
| 20 | "selfcollect*".ab,kw,ti. | 98 |
| 21 | "self-collect*".ab,kw,ti. | 2842 |
| 22 | "selfsampl*".ab,kw,ti. | 86 |
| 23 | "self-sampl*".ab,kw,ti. | 1678 |
| 24 | exp screening/ | 800729 |
| 25 | "screen*".ab,kw,ti. | 1396259 |
| 26 | "early diagno*".ab,kw,ti. | 159762 |
| 27 | "early test*".ab,kw,ti. | 1100 |
| 28 | "early detect*".ab,kw,ti. | 126253 |
| 29 | 20 or 21 or 22 or 23 or 24 or 25 or 26 or 27 or 28 | 1941133 |
| 30 | 19 or 29 | 2270159 |
| 31 | 13 and 30 | 44048 |
| 32 | limit 31 to ((meta analysis or "systematic review") and yr="2014 - Current") | 1184 |

**List of studies included and excluded after full-text analysis**

| Lp. | Authors, Title, Journal | Full text status |
|---|---|---|
| **1** | **Jordaens S.; Zwaenepoel K.; Tjalma W.; Deben C.; Beyers K. Urine biomarkers in cancer detection: A systematic review of preanalytical parameters and applied methods. Int J Cancer. 2023 May 15;152(10):2186-2205.** | **Included** |
| **2** | **Cho, H. W.; Shim, R. S.; Lee, J. K.; Hong J. H. Accuracy of human papillomavirus tests on self-collected urine versus clinician-collected samples for the detection of cervical precancer: a systematic review and meta-analysis. J Gynecol Oncol. 2022 Jan;33(1):e4.** | **Included** |
| **3** | **Bober P.; Firment P.; Sabo J. Diagnostic Test Accuracy of First-Void Urine Human Papillomaviruses for Presence Cervical HPV in Women: Systematic Review and Meta-Analysis. Int J Environ Res Public Health. 2021 Dec 17;18(24):13314.** | **Included** |
| **4** | **Nishimura H.; Yeh P. T.; Oguntade H.; Kennedy C. E.; Narasimhan M. HPV self-sampling for cervical cancer screening: a systematic review of values and preferences. BMJ Glob Health. 2021 May;6(5):e003743.** | **Included** |
| **5** | **Pathak N.; Dodds J.; Zamora J.; Khan K. Accuracy of urinary human papillomavirus testing for presence of cervical HPV: systematic review and meta-analysis. BMJ. 2014 Sep 16:349:g5264.** | **Included** |
| 6 | Kirubarajan A.; Leung S.; Li X.; Yau M.; Sobel M. AO. Barriers and facilitators for cervical cancer screening among adolescents and young people: a systematic review. BMC Women's Health 21, 122 (2021). | Excluded |
| 7 | Zheng R.; Heller D.S. High-Risk Human Papillomavirus Identification in Precancerous Cervical Intraepithelial Lesions. J Low Genit Tract Dis. 2020 Apr;24(2):197-201. | Excluded |
| 8 | Caleia A.I.; Pires C.; Pereira J.D.F.; Pinto-Ribeiro F.; Longatto-Filho A. Self-Sampling as a Plausible Alternative to Screen Cervical Cancer Precursor Lesions in a Population with Low Adherence to Screening: A Systematic Review. Acta Cytol. 2020;64(4):332-343. | Excluded |
| 9 | Nodjikouambaye Z.A.; Adawaye C.; Mboumba Bouassa R.-S.; Sadjoli D.; Belec L. A systematic review of self-sampling for HPV testing in Africa. Int J Gynaecol Obstet. 2020 May;149(2):123-129. | Excluded |
| 10 | Arbyn M.; Peeters E.; Donders G.; De Sutter P.; Tjalma W. Valhudes AO.: A protocol for validation of human papillomavirus assays and collection devices for HPV testing on self-samples and urine samples. J Clin Virol. 2018 Oct:107:52-56. | Excluded |
| 11 | Karisani N.; Aminimoghaddam S.; Kashanian M.; Baradaran H.R.; Moradi Y. AO. Diagnostic accuracy for alternative cervical cancer screening strategies: A systematic review and meta-analysis. Health Care Women Int. 2024;45(3):323-362. | Excluded |
| 12 | Rosberger Z.; Tatar O.; Haward B.; Zhu P.; Griffin-Mathieu G. What Are the Challenges of HPV Testing Implementation in the Prevention of Cervical Cancer? Development and Validation of HPV Testing and Self-Sampling Attitudes and Beliefs Scales. Curr Oncol. 2023 Jan; 30(1): 1206–1219. | Excluded |
| 13 | Zigras T.; Mayrand M.-H.; Bouchard C.; Salvador S.; Eiriksson L. | Excluded |

| | | |
|---|---|---|
| | Canadian Guideline on the Management of a Positive Human Papillomavirus Test and Guidance for Specific Populations. Curr Oncol. 2023 Jun 9;30(6):5652-5679. | |
| 14 | Tin K.N.; Ngamjarus C.; Rattanakanokchai S.; Sothornwit J.; Aue-aungkul A. Interventions to increase the uptake of cervical cancer screening in low- and middle-income countries: a systematic review and meta-analysis. BMC Womens Health. 2023 Mar 23;23(1):120. | Excluded |
| 15 | Costa S.; Verberckmoes B.; Castle P.E.; Arbyn M. AO. Offering HPV self-sampling kits: an updated meta-analysis of the effectiveness of strategies to increase participation in cervical cancer screening. Br J Cancer. 2023 Mar;128(5):805-813. | Excluded |
| 16 | Arbyn M.; Simon M.; de Sanjose S.; Clarke M.A.; Poljak M. Accuracy and effectiveness of HPV mRNA testing in cervical cancer screening: a systematic review and meta-analysis. Lancet Oncol 2022; 23: 950–60. | Excluded |
| 17 | Tatara T.; Wnuk K.; Miazga W.; Switalski J.; Karauda D. AO. The Influence of Vaginal HPV Self-Sampling on the Efficacy of Populational Screening for Cervical Cancer-An Umbrella Review. Cancers (Basel). 2022 Nov 30;14(23):5913. | Excluded |
| 18 | Serrano B.; Ibanez R.; Robles C.; Peremiquel-Trillas P.; de Sanjose S. Worldwide use of HPV self-sampling for cervical cancer screening. Prev Med. 2022 Jan:154:106900. | Excluded |
| 19 | Aimagambetova G; Atageldiyeva K; Marat A; Suleimenova A; Issa T. Comparison of diagnostic accuracy and acceptability of self-sampling devices for human Papillomavirus detection: A systematic review. Prev Med Rep. 2024 Jan 4:38:102590. | Excluded |
| 20 | Andrews J. Optimal triage of HPV-positive test results. DOI: https://dx.doi.org/10.1136/ijgc-2019-ESGO.1102 PT - Conference Abstract | Excluded |
| 21 | Poon M.K.L.; Wong J.P.H.; Li A.T.W.; Manuba M.; Bisignano A. HIV-positive MSM's knowledge of HPV and anal cancer self-sampling: A scoping review. Curr Oncol. 2018 Feb;25(1):e83-e89. | Excluded |
| 22 | Kizior A. Barriers to participation in breast and cervical cancer screening (Analysis of international research). DOI: https://dx.doi.org/10.1111/ajco.13498 PT - Conference Abstract | Excluded |
| 23 | Arbyn M.; Smith S.B.; Temin S.; Sultana F.; Castle P. Detecting cervical precancer and reaching underscreened women by using HPV testing on self samples: Updated meta-analyses. BMJ. 2018 Dec 5:363:k4823. | Excluded |
| 24 | Rees I.; Jones D.; Chen H.; Macleod U. Interventions to improve the uptake of cervical cancer screening among lower socioeconomic groups: A systematic review. Prev Med. 2018 Jun:111:323-335. | Excluded |
| 25 | Bik E.; Bird S.W.; Leon L.E.; Ugalde J.A.; Almonacid D.E. A new sequencing-based assay combining HPV detection and identification with vaginal microbiome analysis. DOI: https://dx.doi.org/10.1097/LGT.0000000000000387 PT - Conference Abstract | Excluded |
| 26 | Yared N.; Horvath K.; Fashanu O.; Zhao R. Optimizing screening for sexually transmitted infections in men using self-collected swabs-a systematic review. Sex Transm Dis. 2018 May;45(5):294-300. | Excluded |
| 27 | Koliopoulos G.; Nyaga V.N.; Santesso N.; Bryant A.; Martin-Hirsch P.P.L. Cytology versus HPV testing for cervical cancer screening in the general population. Cochrane Database Syst Rev. 2017 Aug 10;8(8):CD008587. | Excluded |

| | | |
|---|---|---|
| 28 | Del Mistro A.; Frayle H.; Rizzi M.; Fantin G.; Ferro A. Methylation analysis and HPV genotyping of self-collected cervical samples from women not responding to screening invitation and review of the literature. PLoS One. 2017 Mar 6;12(3):e0172226. | Excluded |
| 29 | Nelson E.J.; Maynard B.R.; Loux T.; Fatla J.; Gordon R. The acceptability of self-sampled screening for HPV DNA: A systematic review and meta-analysis. Sex Transm Infect. 2017 Feb;93(1):56-61. | Excluded |
| 30 | Kelly H.; Mayaud P.; Segondy M.; Pant Pai N.; Peeling R.W. AO. A systematic review and meta-analysis of studies evaluating the performance of point-of-care tests for human papillomavirus screening. Sex Transm Infect. 2017 Dec;93(S4):S36-S45. | Excluded |
| 31 | Meggiolaro A.; Unim B.; Semyonov L.; Miccoli S.; Maffongelli E. The role of Pap test screening against cervical cancer: a systematic review and meta-analysis. Clin Ter. 2016 Jul-Aug;167(4):124-39. | Excluded |
| 32 | Elfstrom K.M.; Arnheim-Dahlstrom L.; Von Karsa L.; Dillner J. AO. Cervical cancer screening in Europe: Quality assurance and organisation of programmes. Eur J Cancer. 2015 May;51(8):950-68. | Excluded |
| 33 | Verdoodt F.; Jentschke M.; Hillemanns P.; Racey C.S.; Snijders P.J.F. Reaching women who do not participate in the regular cervical cancer screening programme by offering self-sampling kits: A systematic review and meta-analysis of randomised trials. Eur J Cancer. 2015 Nov;51(16):2375-85. | Excluded |
| 34 | Albrow R.; Blomberg K.; Kitchener H.; Brabin L.; Patnick J. Interventions to improve cervical cancer screening uptake amongst young women: A systematic review. Acta Oncol. 2014 Apr;53(4):445-51. | Excluded |
| 35 | Arbyn M.; Verdoodt F.; Snijders P.J.F.; Verhoef V.M.J.; Suonio E. Accuracy of human papillomavirus testing on self-collected versus clinician-collected samples: A meta-analysis. Lancet Oncol. 2014 Feb;15(2):172-83. | Excluded |
| 36 | Mekuria S.F.; Timmermans S.; Borgfeldt C.; Jerkeman M.; Johansson P. HPV self-sampling versus healthcare provider collection on the effect of cervical cancer screening uptake and costs in LMIC: a systematic review and meta-analysis. Syst Rev. 2023 Jun 22;12(1):103. | Excluded |
| 37 | Nwaozuru U; Obiezu-Umeh C; Obi-Jeff C; Shato T; Gbaja-Biamila T. A systematic review of randomized control trials of HPV self-collection studies among women in sub-Saharan Africa using the RE-AIM framework. Implement Sci Commun. 2021; 2: 138. | Excluded |

## AMSTAR2

The systematic reviews included in the analysis received the following ratings:

- high – Pathak 2014
- low – Cho 2022, Nishimura 2021
- critically low – Joardens 2023, Bober 2021

| Publication | Item 2 | Item 4 | Item 7 | Item 9 | Item 11 | Item 13 | Item 15 | Overall rating |
|---|---|---|---|---|---|---|---|---|
| Joardens 2023 (SR) | Partial Yes | Partial Yes | No | No | No meta-analysis conducted | No | No meta-analysis conducted | **Critically low** |

| Publication | Item 2 | Item 4 | Item 7 | Item 9 | Item 11 | Item 13 | Item 15 | Overall rating |
|---|---|---|---|---|---|---|---|---|
| Cho 2022 (MA) | Partial Yes | Partial Yes | No | Partial Yes | Yes | Yes | Yes | **Low** |
| Bober 2021 (MA) | No | Partial Yes | No | Partial Yes | Yes | Yes | Yes | **Critically low** |
| Nishimura 2021 (SR) | Partial Yes | Partial Yes | No | Partial Yes | No meta-analysis conducted | Yes | No meta-analysis conducted | **Low** |
| Pathak 2014 (MA) | Yes | Patrial Yes | Patrial Yes | Partial Yes | Yes | Yes | Yes | **High** |

*MA – meta-analysis; SR – systematic review*

*Critical domains: item 2 – protocol registered before commencement of the review; item 4 – adequacy of the literature search; item 7 – justification for excluding individual studies; item 9 – risk of bias from individual studies being included in the review; item 11 – appropriateness of meta-analytical methods; item 13 – consideration of risk of bias when interpreting the results of the review; item 15 – assessment of presence and likely impact of publication bias.*
